# Unveiling the Enigmatic: A Rare Presentation of Posterior Reversible Encephalopathy Syndrome in a Primigravida With Twin Pregnancy

**DOI:** 10.7759/cureus.48443

**Published:** 2023-11-07

**Authors:** Swasti Shukla, Deepti Shrivastava

**Affiliations:** 1 Obstetrics and Gynaecology, Jawaharlal Nehru Medical College, Acharya Vinoba Bhave Rural Hospital, Datta Meghe Institute of Higher Education and Research, Wardha, IND

**Keywords:** clinical heterogeneity, neuroimaging, cerebral edema, seizures, pregnancy, posterior reversible encephalopathy syndrome (pres)

## Abstract

Posterior reversible encephalopathy syndrome (PRES) is a distinctive and challenging neurological condition characterized by a constellation of symptoms, including altered mental status, seizures, headaches, and visual disturbances. It is often associated with abrupt increases in blood pressure or other underlying precipitating factors. While PRES has been recognized for its diverse clinical presentations, it remains an infrequent diagnosis, and its occurrence during pregnancy, especially in primigravida with multiple gestations, is rare. In this context, it is imperative to explore and explicitly mention the underlying factors contributing to PRES in the case, which may include factors such as hypertensive disorders of pregnancy, immunosuppressive therapy, and renal dysfunction. Addressing these factors is essential for a comprehensive understanding of PRES in the context of pregnancy and its implications for clinical management. In this case report, we present an unusual and captivating clinical scenario involving a 19-year-old primigravida admitted to a tertiary care hospital with a twin pregnancy and presenting with complaints of severe back pain and a history of amenorrhea for eight months. The patient's journey unfolds with an emergency cesarean section, resulting in the birth of two healthy female infants and the sudden onset of seizures on the second day postoperatively. This case provides an intriguing glimpse into the complexities of diagnosing and managing PRES, particularly within the unique context of pregnancy. We discuss the clinical course, diagnostic evaluation, and the subsequent management of this challenging case, contributing to the growing body of knowledge on PRES in a pregnancy-related setting.

## Introduction

Posterior reversible encephalopathy syndrome (PRES), first described by Hinchey et al. in 1996 [1], represents a distinctive and enigmatic neurological condition characterized by a spectrum of clinical manifestations, including altered mental status, seizures, headaches, and visual disturbances [1]. Although PRES is increasingly acknowledged, its diagnosis remains a relatively uncommon occurrence, and its presentation during pregnancy, particularly in primigravida with multiple gestations, continues to be a rare and intriguing clinical scenario [2].

Hinchey's pioneering work emphasized the importance of understanding PRES as a clinico-radiological entity, where characteristic neuroimaging findings are pivotal in its diagnosis [2]. The syndrome is often associated with acute hypertension or other precipitating factors, which can lead to cerebral edema, predominantly affecting the posterior regions of the brain [3]. It is pertinent to elaborate on these predisposing factors, which may encompass various elements such as hypertensive disorders of pregnancy, immunosuppressive therapy, renal dysfunction, autoimmune diseases, or cytotoxic drugs [4]. Despite the growing awareness of PRES, its clinical heterogeneity and the rarity of its occurrence in pregnancy continue to pose diagnostic and management challenges [4].

## Case presentation

A 19-year-old female was brought to the emergency department of a tertiary care hospital with the chief complaint of severe back pain. Upon receiving the patient, a comprehensive medical history was obtained from her husband. It was revealed that the back pain had suddenly intensified, prompting them to rush to the hospital. Additionally, the patient had a history of amenorrhea for the past eight months, which had initially raised concerns about her reproductive health. This unique medical history became more significant when it was discovered that she was a primigravida with a twin pregnancy. Notably, she had experienced a prior hospitalization at the 19th week of gestation for cervical stitch placement, which marked a critical milestone in her pregnancy journey. The mention of her pregnancy history serves to underscore the context and significance of her presenting complaints, as it may have relevance to the subsequent development of her medical condition.

Following a thorough physical examination and obtaining consent from both the patient and her husband, the decision was made to transfer the patient for an emergency cesarean section. The patient subsequently underwent an emergency lower segment cesarean section (LSCS) under spinal anesthesia, resulting in the birth of two live female children weighing 1.750 kg and 2.010 kg, respectively. The infants cried immediately after birth, and the patient was subsequently moved to the recovery room for postoperative observation.

**Figure 1:**
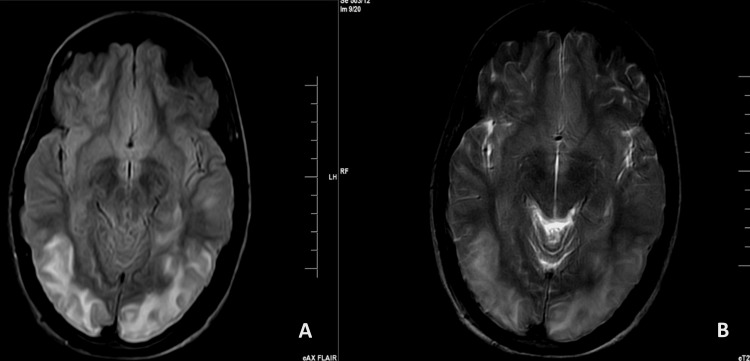
FLAIR MRI (A) and T2WI MRI (B) image axial section showing hyperintense areas in the bilateral temporo-occipital regions FLAIR: Fluid-attenuated inversion recovery; T2WI: T2 weighted image

On the second day post-LSCS, the patient experienced a sudden onset of seizures despite having no history of seizure disorder since birth. It is vital to note that the patient's blood pressure levels throughout this presentation were monitored closely. The seizures were managed through intravenous administration of Inj. MgSO_4_ according to the Pritchard regimen, followed by Inj. levetiracetam 1 gm stat and 500 mg every 12 hours. There was no history of seizure disorder in the patient's family. The patient was referred to a neurologist, who conducted a neurological examination and recommended a magnetic resonance imaging (MRI) scan. The MRI revealed altered signal intensity in the bilateral temporal-occipital parietal lobes and the left cerebellar hemisphere, with noted right inferior turbinate hypertrophy (Figure 1). Based on these findings, the neurologist diagnosed the patient with PRES and recommended treatment with lacosamide 200 mg stat intravenous, followed by 100 mg every 12 hours. Tracking the patient's blood pressure levels during the presentation is crucial in understanding the clinical context and management of PRES, particularly in the absence of a prior seizure history.

## Discussion

The case presented here offers a captivating glimpse into the intriguing clinical complexities of PRES within the unique context of a primigravida with a twin pregnancy. As discussed earlier, the patient's journey from severe back pain to developing seizures post-LSCS presented a diagnostic challenge. It underscored the importance of recognizing PRES in this population.

Pregnancy-associated PRES is rare yet noteworthy, and its pathophysiology remains incompletely understood. While the exact mechanisms are not entirely clear, it is believed that the hemodynamic changes, endothelial dysfunction, and hormonal fluctuations during pregnancy may predispose individuals to PRES [4]. However, what makes this case particularly exceptional is introducing a novel and less common precipitating factor to PRES than the traditional high blood pressure, adding an extra layer of complexity to its etiology.

The patient's history of amenorrhea, a twin pregnancy, and a prior cervical stitch placement at 19 weeks introduced a unique dimension to the presentation. In this case, the abrupt onset of seizures is a hallmark of PRES, but it is not always present. Neurological symptoms can range from headaches and altered mental status to visual disturbances and focal neurological deficits [5]. This emphasizes the importance of including PRES in differential diagnoses when patients present with sudden neurological disturbances, particularly in obstetric cases.

The diagnosis of PRES can be elusive, and in the context of pregnancy, the challenge is compounded [6]. The emergence of seizures in a postoperative setting after LSCS led to an initial consideration of eclampsia, highlighting the diagnostic dilemma [7]. Neuroimaging, particularly MRI, was pivotal in confirming the diagnosis by revealing the characteristic altered signal intensity in the bilateral temporal-occipital parietal lobes [8]. This reinforces the significance of advanced neuroimaging techniques in differentiating PRES from other neurological conditions [9].

The management of PRES primarily revolves around the prompt control of blood pressure, often with antihypertensive medications. Our patient responded well to intravenous MgSO_4_, levetiracetam, and lacosamide, with the cessation of seizures and improved neurological status. Timely recognition and intervention are paramount in mitigating the potential complications associated with PRES, including permanent neurological deficits [10]. This case underscores the need for healthcare providers to maintain a high index of suspicion for PRES when patients, especially pregnant individuals, present with unexplained neurological symptoms. Understanding the diverse clinical scenarios PRES can manifest is critical, as delayed diagnosis and treatment may have adverse consequences. By sharing this case, we aim to contribute to the growing body of knowledge regarding PRES and raise awareness among healthcare professionals.

## Conclusions

This case report offers a profound insight into the unique and intricate presentation of PRES in a primigravida with a twin pregnancy. The patient's journey, marked by severe back pain, amenorrhea, and a history of cervical stitch placement, culminated in an emergency cesarean section. The emergence of seizures postoperatively, despite the absence of a prior seizure disorder, posed a perplexing development. The diagnostic process, encompassing neurological evaluation and MRI, ultimately led to the identification of altered signal intensity in the brain and the diagnosis of PRES. This uncommon manifestation of PRES during the peripartum period emphasizes the necessity for heightened awareness of the syndrome in such clinical contexts. Moreover, it underscores the importance of comprehensive clinical assessment. The implications of delayed recognition of PRES lie in the potential for detrimental consequences. When PRES is not promptly identified, its clinical manifestations can worsen, leading to an exacerbation of symptoms and an increased risk of complications. These complications can include permanent neurological deficits, which can profoundly impact the patient's quality of life. In this context, delayed recognition can result in a less favorable prognosis and hinder the patient's recovery. Therefore, this case underscores the significance of considering PRES as a differential diagnosis in patients presenting with perplexing neurological symptoms, particularly in the peripartum period. By sharing this case, we aim to contribute to the expanding knowledge base on PRES and enhance healthcare providers' awareness of its potential occurrence in diverse clinical settings. Timely diagnosis and intervention are paramount, as they are essential for improving patient outcomes and mitigating the risk of lasting neurological sequelae.
